# Phylogeography of the *Alcippe morrisonia *(Aves: Timaliidae): long population history beyond late Pleistocene glaciations

**DOI:** 10.1186/1471-2148-9-143

**Published:** 2009-06-27

**Authors:** Gang Song, Yanhua Qu, Zuohua Yin, Shouhsien Li, Naifa Liu, Fumin Lei

**Affiliations:** 1 Key Laboratory of Zoological Systematics and Evolution, Institute of Zoology, Chinese Academy of Sciences, Beijing, 100101 PR China; 2 Graduate School of the Chinese Academy of Sciences, Beijing, 100039 PR China; 3 Department of Life Science, National Taiwan Normal University, Taibei, Taiwan, 116 ROC; 4 School of Life Science, Lanzhou University, Lanzhou, 730000 PR China

## Abstract

**Background:**

The role of Pleistocene glacial oscillations in current biodiversity and distribution patterns varies with latitude, physical topology and population life history and has long been a topic of discussion. However, there had been little phylogeographical research in south China, where the geophysical complexity is associated with great biodiversity. A bird endemic in Southeast Asia, the Grey-cheeked Fulvetta, *Alcippe morrisonia*, has been reported to show deep genetic divergences among its seven subspecies. In the present study, we investigated the phylogeography of *A. morrisonia *to explore its population structure and evolutionary history, in order to gain insight into the effect of geological events on the speciation and diversity of birds endemic in south China.

**Results:**

Mitochondrial genes cytochrome b (Cytb) and cytochrome c oxidase I (COI) were represented by 1236 nucleotide sites from 151 individuals from 29 localities. Phylogenetic analysis showed seven monophyletic clades congruent with the geographically separated groups, which were identified as major sources of molecular variance (90.92%) by AMOVA. TCS analysis revealed four disconnected networks, and that no haplotype was shared among the geographical groups. The common ancestor of these populations was dated to 11.6 Mya and several divergence events were estimated along the population evolutionary history. Isolation by distance was inferred by NCPA to be responsible for the current intra-population genetic pattern and gene flow among geographical groups was interrupted. A late Pleistocene demographic expansion was detected in the eastern geographical groups, while the expansion time (0.2–0.4 Mya) was earlier than the Last Glacial Maximum.

**Conclusion:**

It is proposed that the complicated topology preserves high genetic diversity and ancient lineages for geographical groups of *A. morrisonia *in China mainland and its two major islands, and restricts gene exchange during climate oscillations. Isolation by distance seems to be an important factor of genetic structure formation within geographical populations. Although glacial influence to population fluctuation was observed in late Pleistocene, it seems that populations in eastern China were more susceptible to climate change, and all geographical groups were growing stably through the Last Glacial Maximum. Coalescence analysis suggested that the ancestor of *A. morrisonia *might be traced back to the late Miocene, and the current phylogeographical structure of *A. morrisonia *is more likely to be attributable to a series geological events than to Pleistocene glacial cycles.

## Background

The glacial cycles of the past two million years have traditionally been considered to have had profound effects on the genetic patterns of most extant species [1-3]. However, this paradigm has been long and hotly debated [4-6]. Some studies contend that the late Pleistocene was an important time for avian evolution, involving phylogeographic separations and the completion of speciation events [1,7,8]. Other studies, in contrast, suggest that the speciation events could be projected back to the Miocene or even earlier times, and speciation was not accelerated by the later glacial oscillation [9-11]. Even most intraspecific separations, commonly considered to be footprints of the late Pleistocene climate shifts, were found to have been initiated at least one million years ago [6]. On the other hand, it is apparent that the impact of the Pleistocene ice age on phylogeography and speciation depended on latitude and topography, and varied with population life history and geography [3]. On the basis of phylogeographic comparisons of North American songbirds, Zink found that co-distributed species had different evolutionary histories [12]. Weir & Schluter compared the timing of speciation events from the boreal zone to the Neotropic and found a strong latitudinal trend of speciation time. These results showed that most boreal speciation events were related to the Pleistocene ice age, while splits of Neotropic species decreased at that time [13]. All these discussions and debates call for further phylogeographic studies to complement our knowledge about the genetic footprints of the ice age. However most studies have been done in Europe and North America, comparative information across different regions of the globe needs to be synthesized to elucidate the effects of Pleistocene climate shifts on the formation of present-day diversity.

As one of the species richness hotspots in Southeast Asia, south China has high biodiversity [14-16], reflecting its high topological complexity [17]. Several high mountains wind through this region, such the Hengduan, Qinling and Nanling Mountains. Two main islands, Taiwan and Hainan, have been repeatedly connected and disconnected with the mainland during the past million years [18]. It has been proposed that the topographical complexity of this region would provide stable habitats during an ice age, where species could survive in different refugia and new lineages would be generated [2]. However, little research has focused on south China. Two recent studies on herpetological phylogeography revealed intraspecific divergence, bottlenecks and demographic expansion as a result of the late Pleistocene climate changes [19,20]. No other phylogeographic studies on terrestrial vertebrates in this region exist to our knowledge. The sparse literature on this region limits our understanding of the formation of high diversity within the complicated topography of south China.

The *Alcippe morrisonia *is a small babbler widely distributed in tropical and subtropical habitats from Burma to Taiwan [21]. It is a dominant species, with foraging flocks in the medium and understory of tropical rain forest and subtropical broadleaf evergreen forest [22,23]. As a typical Oriental species, all seven subspecies can be found in the upland and lowland parts of south China [24]. Recent studies on the molecular taxonomy of *A. morrisonia *and its relatives have shown that the genus *Alcippe *is a polyphyletic group [25]. The complicated intraspecific relationships are detected within *A. morrisonia*, in which *Alcippe peracensis annamensis *was grouped within *A. morrisonia *[26]. Another study on the phylogenetic relationships of the subspecies of *A. morrisonia *using the complete ND2 gene confirmed this taxonomic complexity and found deep genetic divergences among subspecies [27]. The peculiarly deep genetic divergences within a traditional "species" identified by morphological characters raise questions about how this pattern came into being. In this study, therefore, we examine the phylogeography of *A. morrisonia *and explore its possible mechanisms responsible for the current genetic pattern to provide an insight into the ice age legacy in south China.

## Results

### Phylogenetic analysis

**Figure 1:**
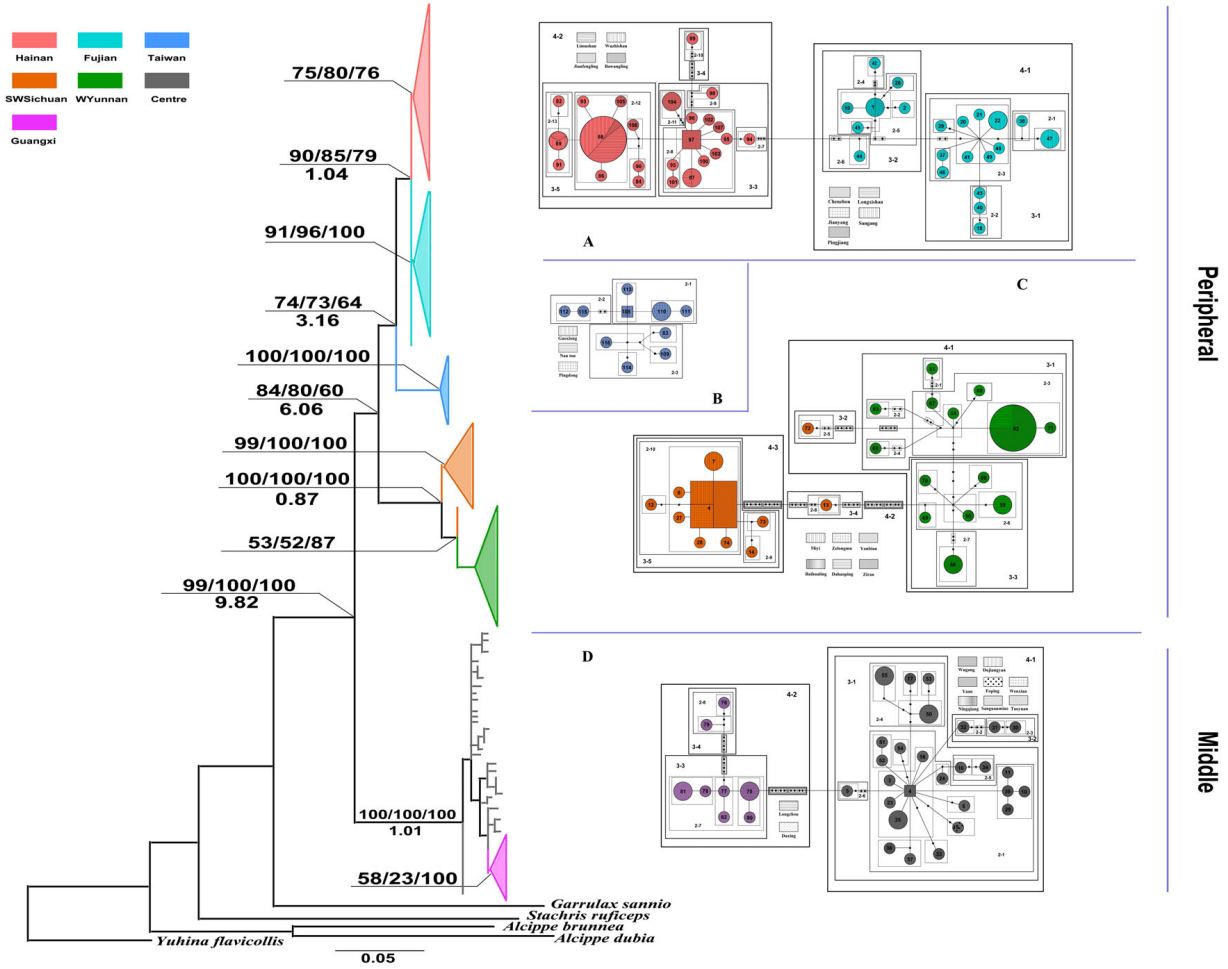
**Maximum-likelihood tree and nested clade TCS networks based on combined dataset**. Nodal values above the line indicate bootstrap supports and poster probabilities of MP/ML/BI, while the values under the line are the divergence times estimated by MDIV. Colours represent geographical groups and spatterworks stand for locations of sample sites. The black dots refer to missing steps intermediate between observed haplotypes. Nested clades are indicated by 'N-#', where N is the nesting level and # is the number of individuals assigned to the clades within each level.

We obtained 642 bp of the partial Cytb gene from 156 individuals and 594 bp of the partial COI gene from 153 individuals. The Cytb sequences yielded 141 variable sites (118 were parsimony informative), identifying 88 haplotypes (GenBank Access Number FJ472657–FJ472744), and the COI sequences contained 99 variable sites of which 81 were parsimony informative, generating 71 haplotypes (GenBank Access Number FJ472745–FJ472815). Modeltest indicated that the best substitution models were HKY+G and TIM+I for Cytb and COI respectively. For the combined sequence data, a total of 151 sequences of 1236 bp were obtained, as some individuals could not be sequenced for both partial genes. For the combined sequence data set, there were 239 variable sites of which 199 were parsimony informative, generating 116 haplotypes. The best model for the combined dataset was TrN+I+G. Phylogenetic trees estimated by three methods (MP, ML and BI) for haplotypes of Cytb, COI and the combined dataset were generally compatible, the differences being the relative positions and the statistical support possibilities of some branches, then the topology based on the combined dataset is presented in Figure 1. The tree is geographically structured and haplotypes from neighbouring locations are mostly clustered. Seven geographic groups were identified: Fujian, Hainan, Taiwan WYunnan, SWSichuan, Centre and Guangxi. Most of the locations of these geographical phylogroups were consistent with the subspecies distribution ranges. However, some haplotypes are misplaced, such as H72, which clustered into the WYunnan group though its sampling sites are in SWSichuan. The monophyly of the Fujian and Centre groups was ambiguous. Two major clades with high support probabilities were confirmed: the "Peripheral" clade contains haplotypes from Fujian, Hainan, Taiwan, WYunnan and SWSichuan groups; the "Middle" clade consists of haplotypes from Centre and Guangxi groups.

### Population genetic structure

All analyses for population genetics and demographic history were based on the combined sequences. Ninety-five of the 116 haplotypes generated by the combined dataset were singletons. Among the other 21 shared haplotypes, seven were shared between two or three neighbouring localities (Additional file 1). No haplotype was shared among the geographical groups. AMOVA showed that most of the variance came from differences among groups, and the seven groups were best recognized because the grouping maximized the values of among-group variance (90.92%), while 8.77% of the variance from differences among individuals (Additional file 2). Most geographical groups had high haplotype diversities (0.9532–0.9935) except for SWSichuan (0.8304). High nucleotide diversities were observed in Fujian (0.54%) and WYunnan (0.65%), while those in the two insular groups, Hainan and Taiwan, were relatively low (0.33% and 0.31%) (Additional file 3).

Analysis of TCS yielded four unconnected haplotype networks, which were concurrent with the topology described in the phylogenetic tree (Figure 1). In networks A, C and D, haplotypes from the locations within the same geographical group linked to each other and then connected with haplotypes in the neighbouring group. Unexpectedly, haplotypes from the Fujian group were connected with those from Hainan rather than Taiwan, while haplotypes from Taiwan formed an isolated clade, network B. In network C, haplotype H72 from SWSichuan was linked to the WYunnan group by thirteen mutations. More than five mutations were observed between two haplotypes within networks A, C and D. Some phylogeographical structures were detected within clades. Allopatric fragmentation was found in network A. For low level clades, restricted gene flow with isolation by distance was revealed (clades 2–12, 4-1, 4-2 in network A; clade 2-1 in network D). But these associations were not confirmed by the Mantel Test or IBDWS; only clade 4-1 in network A showed a tendency towards a significant IBD pattern (*P *= 0.08) (Additional file 4).

### Population demographic history

The average net distance between geographical groups was 0.060 for the combined sequence and 0.067 for Cytb alone. We selected a conventional mutation rate for the avian mitochondrial cytochrome b gene in our study (1.00*10^-8 ^per site per year) and multiplied it by a factor of 0.90 to reflect the mutation rate of the combined sequence. The times of divergence between Fujian and Hainan, WYunnan and SWSichuan, Centre and Guangxi were 1.15, 0.87 and 1.12 million years ago, respectively. The Taiwan group separated from the Fujian and Hainan groups at 3.51 Mya, and the divergence time of the western (WYunnan and SWSichuan) and eastern (Fujian, Hainan and Taiwan) groups was 6.06 Mya. The divergence time between the Middle and Peripheral clades was earlier, dating back to 9.82 Mya (Figure 1). Restricted gene flows were found between all geographical group pairs, and the maximum M value (0.12) occurred between WYunnan and SWSichuan. The time of the most recent common ancestor (t_mrca_) for all haplotypes of *A. morrisonia *was estimated back to 11.66 Mya (Table 1).

**Table 1:** Time estimation of demographical history of *A. morrisonia *by MDIV and Bayesian skyline plot methods

	**MDIV**				**Bayesian skyline plot**	
			
**group pairs**	***M***	***t*_*div *_**(Mya)	***t*_*mrca *_**(Mya)	**Groups**	***t*^*a *^since expansion (Mya)**	***t*_*mrca *_(Mya)**
				Fujian	0.20	0.51
Fujian-Hainan	0.02	1.15	1.72	Hainan	0.15	0.44

Fujian/Hainan-Taiwan	0.02	3.51	4.81	Taiwan	0.10	0.37
				Wyunnan	0.30	0.67

WYunnan-SWSichuan	0.12	0.87	2.87	SWSichuan	0.23	0.76
				Centre	0.17	0.27

Centre-Guangxi	0.06	1.12	1.73	Guangxi	/	0.17

Fujian/Hainan/Taiwan-WYunnan/SWSichuan	0.04	6.06	8.03	Peripheral	/	7.63
				Middle	/	0.76
Peripheral-Middle	0.04	9.82	11.60	Total	/	8.11

*M*, scaled migration rate, and M = 2Nef * m, *T*_*mrca*_, the time of MRCA in units of mutation rate and *t*_*mrca*_, the geological time transformed from *T*_*mrca *_by the mutation rate 0.9 * 10^-8^. *t*^*a*^, time since expansion, /, no demographic expansion was detected.

**Figure 2:**
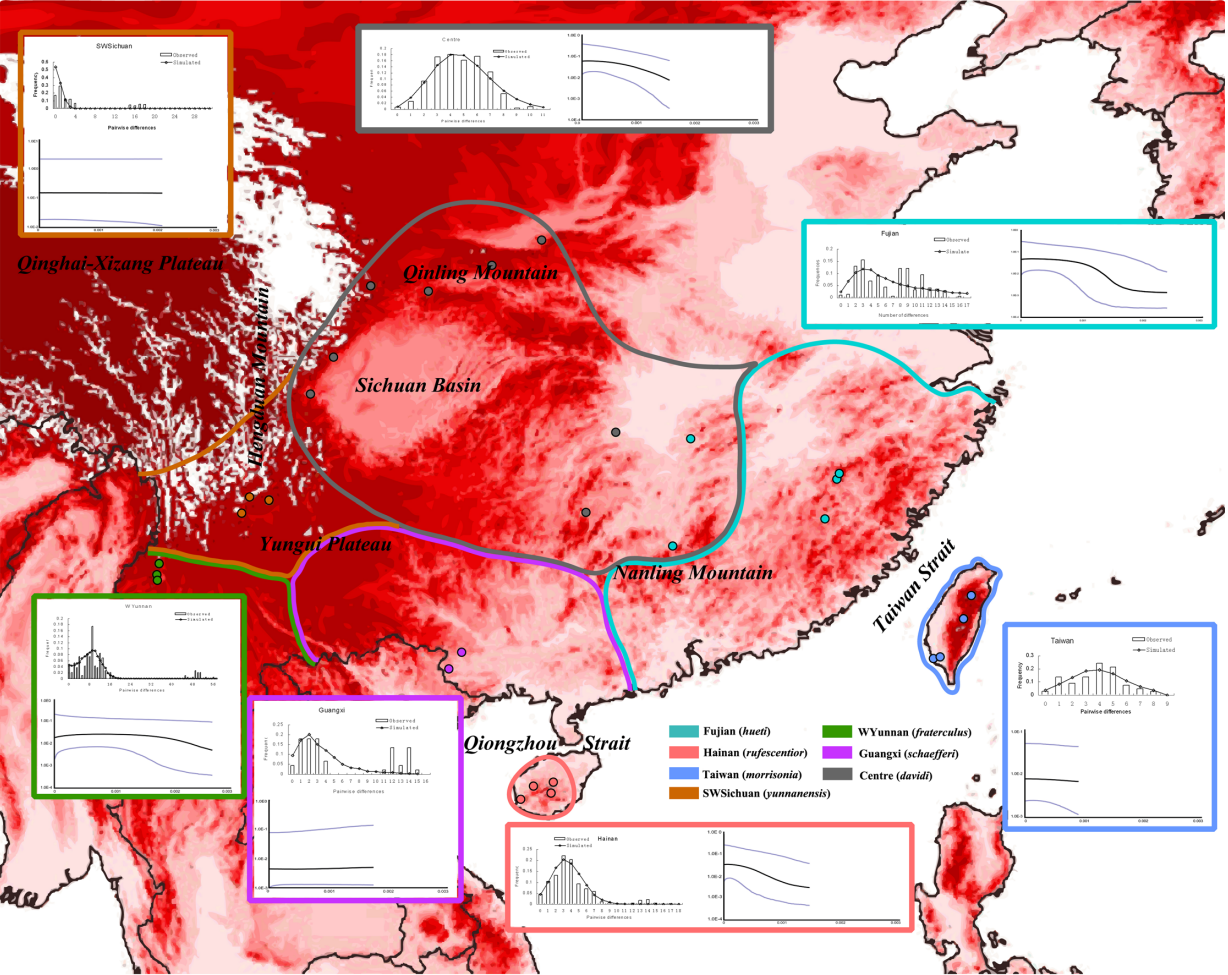
**Mismatch distribution and Bayesian skyline plot for geographical groups of *A. morrisonia***. Coloured dots stand for phylogenetic relationships of sampling sites and colour boundaries indicate the distribution ranges of each subspecies of *A. morrisonia *according to Cheng et al. The histograms in the MD represent the observed frequencies of pairwise differences among haplotypes and the line shows the curve expected for a population that has expanded. The X axis in the BSP represent numbers of mutations and the Y axis is Ne*μ (effective population size * mutation rate per generation). Italic characters label the main geographical barriers in South China.

Tajima's *D *showed significant negative values in Hainan, SWSichuan and Centre, indicating significant differences from expectation under neutrality, and Fu's *F*_*s *_test showed significant negative values in the Fujian, Hainan, Taiwan and Centre groups (Additional file 3). The demographical dynamics of the seven geographical groups were inferred from mismatch distributions. The results showed that the mismatch distributions in Fujian, Hainan, Taiwan, WYunnan and Centre groups fitted unimodal curves (Figure 2). The variances (SSD) and Harpending raggedness indices indicated that the curves did not differ significantly from the distributions expected under the model of population expansion. With the 0.9 * 10^-8 ^corrected mutation rate and a generation time of two years, the estimated times since population expansion for Fujian, Hainan, Taiwan, and Centre were 0.18, 0.33, 0.39, and 0.43 Mya respectively, corresponding to the penultimate glacial cycle in the late Pleistocene (Table 1). The expansion time for the WYunnan group was earlier than for the other groups with a value of 0.81 Mya. SWSichuan and Guangxi did not fit the demographical expansion model according to the MMD shapes and Fu's neutrality tests.

The Bayesian skyline plot (BSP) simulated the fluctuation of populations over time. Recent population increases were observed in the Fujian, Hainan, WYunnan and Centre groups; the times of growth were estimated at 0.20, 0.15, 0.30, and 0.23 Mya respectively (Figure 2; Table 1). The population sizes of the other three groups have remained rather stable over time. The time of most recent common ancestor for all the haplotypes was dated to 8.11 Mya (Table 1).

## Discussion

### Biogeography and evolutionary history of Alcippe morrisonia

Phylogenetic reconstruction shows that populations from the middle of south China (Centre & Guangxi) are rooted at the base of the tree. Although geographically separated, the eastern populations (Fujian, Hainan and Taiwan) are sister groups of the western populations (WYunnan and SWSichuan), forming the Peripheral clade. The estimated T_mrca _of all haplotypes of *A. morrisonia *could be tracked back to 11.6 Mya by MDIV and to 8.11 Mya by BEAST, which is located in the late Miocene [28]. The first population differentiation between the Peripheral and Middle clades was estimated at 9.82 Mya. Other population divergences with different time scales were detected by MDIV: three pairs of geographical groups were separated about 0.87–1.15 Mya, while the divergence time between the Taiwan group and its continental relatives was 3.51 Mya, and two subclades (eastern and western populations) within the Peripheral clade separated after 6.06 Mya. We propose here that *A. morrisonia *originated in the late Miocene and colonized a wide area from the centre of south China to the peripheral regions. The possible causes of the first population split were environmental shifts due to the uplift of the Tibetan Plateau at the end of the Miocene [29,30]. Although the isolation by distance pattern cannot be rejected, the phylogenetic result suggests that certain geological events and geographical barriers are more likely related to the population structure coming into being. For example, if the population structure was formed under isolation by distance scenario, the genetic distances of SWSichuan to Centre and Fujian to Centre would be smaller than that of SWSichuan to Fujian. In fact SWSichuan was more related to Fujian than to Centre. During the period within a few million years of 8 Mya, a wide variety of changes took place in the region surrounding and including the rise of the Tibetan Plateau. This episode of fauna turn over in Mio-Pliocene boundary was also evidenced by the rodent fossils from Northern Pakistan [31,32]. Environmental changes in much of eastern Asia were suggested to either become drier or precipitation become more seasonally concentrated [33]. It is reasonable to assume that environmental changes in south China with a significant rise of the Tibetan Plateau account for the two major clades (Peripheral and Middle) differentiation of *A. morrisonia *around 8 Mya. The other two subsequent divergences ascended to 6.06 and 3.51 Mya respectively, are presumably consequences of the global substitution of C3 to C4 vegetation [34], coinciding with the major diversifications of the birds of genus *Garrulax *[35], which are closely related to *A. morrisonia*. The three recent population divisions were traced back to the Sicilian Stage in the early Pleistocene (MIS 20-MIS 30, 0.8–1.0 Mya) [36], consistent with the dramatic climate shifts called the "Middle Pleistocene Revolution", during which the Milankovitch oscillation changed from 40,000 to 100,000 years [37]. Taking these arguments together, we suggest that the chronology of genetic divergence in the *A. morrisonia *might be the result of various geological events.

There is no obvious trend of genetic diversity for geographical groups along the lat/longitude gradient. High haplotype and nucleotide diversity was observed both in western and in eastern geographical groups in the China continent. Deep genetic gaps and restricted gene flow were identified among the geographical groups. The interruption of gene flow along geographical groups of *A. morrisonia *is quite different from another phylogeographical study based on Chinese Hwamei (*Leucodioptron canorum canorum*) [38], which has a similar distribution range as *A. morrisonia*'s in China continent. Putative gene flow was observed among geographical groups of *L. c. canorum *while it was hardly detected in *A. morrisonia*. We consider that some of the causes might be related to the limited gene exchange among geographical groups. Firstly, geographical barriers seem to be important effects on population divergences. Great mountains and deep valleys in south China possibly shaped multi-refugia for *A. morrisonia *in the cold weather extension, and then blocked secondary contact during the postglacial recolonization. It can be imagined that the complicated topology of this region played an important role in initiating phylogeographic differentiation and further sculpting pre-existing phylogeographic variety during the glacial oscillations. Secondly, as a sedentary bird in the forest understory and shrubs, small size and poor dispersal capability [39] of *A. morrisonia *might impede gene flow among populations with a distant range, especially in the complicated topology of south China.

Within the geographical groups, association of restricted gene flow with isolation by distance was inferred from NCPA analysis, supposing that geographical distance was an important factor in forming the current genetic structure. This is consistent with the moderate mobility and limited foraging range of *A. morrisonia *[40] and the physical topology of south China. However, the scenario was not well supported by the results of Mantel test and IBDW analysis at the same clade level. Possible causes for this discrepancy might lie in either the haplotype difference involved in the three methods or our limited spotty sampling. NCPA has long been debated for its validity and the risk of false-positives [41,42]. With a difficult population structure to study and having no better method to apply to our analysis, we nevertheless accept the outcome here cautiously. Dense samples from more sites in its distribution range might improve our understanding and make it more reliable.

Two insular populations, Hainan and Taiwan, were separated from the mainland by the strait barriers, while divergence times of the two insular groups from their continental relatives are discordant (1.15 and 3.51 Mya respectively). This is similar to the genetic variance of Chinese mainland Hwamei with their two island relatives [43]. A possible cause for the difference in divergence times between Hainan and Taiwan Islands might be ecological barriers. Both islands repeatedly connected and disconnected with the continent during the Pleistocene, and the last separation happened around 10,000 years ago [18]. Although a large land surface emerged between Taiwan and the mainland with sea level retreat, it is supposed that the flora covering this area was temperate deciduous broad-leaved forest and steppe rather than evergreen broad-leaved forest during the glaciations [44]. Absence of appropriate habitat may have constricted gene flow between the Taiwan and mainland populations in spite of the connection. Hainan Island is close to the tropic, and the strait interval is just 29.5 km wide. The homogeneity of the vegetation probably kept contact between the geographical groups after the sea level fell. Thus, the genetic distance between Fujian and Hainan is much smaller than between Fujian and Taiwan. Analysis of diversity and distribution patterns of endemic birds in China found Taiwan Island has more endemic species than Hainan, which was supposed a result of earlier isolation from mainland of Taiwan than Hainan [45,46]. Here we suggest that the ecological barriers might be more plausible to the different divergence of two insular islands from China mainland.

### Recent population expansion and the late Pleistocene paleoenvironment

Paleovegetation based on pollen data implied that large areas of the exposed continental shelf in eastern China might have been dominated by grasslands, while the uplands of South China were occupied by less dense coniferous or temperate forests during glacier extension [47,48]. There is a contrary opinion that subtropical broadleaved evergreen forest is more plausible as the succession of rain forest during the glacial maximum [49]. As a typical bush babbler, *A. morrisonia *mostly inhabits shrub, the understory of the tropical and sub-tropical evergreen leaf forests, so its demographical fluctuation might reflect changes in the flora of south China. Significant negative Tajima's D and Fu's Fs values were detected in the Fujian, Hainan, Taiwan and Centre groups, and mismatch distribution showed that most groups fit population expansion except for SWSichuan. Recent demographic expansions were also found in the eastern geographical groups by Bayesian skyline plot reconstruction, while the western groups WYunnan, SWSichuan and Guangxi seemed stable in the late Pleistocene. It seems that expansions occurred in the eastern groups rather than in the west, suggesting intense changes of flora in the eastern part of south China. Compared with the continuous high mountains in the western part of south China, the mountains in the east are isolated and low, so the vegetation communities might have changed more drastically in the eastern part of south China during the glacial oscillation [50,51]; demographical expansions were therefore more evident in the eastern groups than in the west.

The time since demographical expansion was estimated mostly around 0.2–0.4 Mya, earlier than the Last Glacial Maximum (LGM), which was regarded as a vital event inducing population divergence [52-54]. Inconsonantly, the present results suggest that great vegetation changes most probably occurred during the largest glacial extension stages in the late Pleistocene, dating in the Marine Isotope Stages 16–18 (MIS 16-MIS 18, 0.6–0.7 Mya). Previous paleoclimates based on δ^18 ^O value and pollen data evidenced that the most substantial glacial extension occurred MIS 16-MIS 18 in China [55]. Since then, environmental changes seem to be moderate in subsequent climate oscillations in the eastern part of south China, where populations were growing stably throughout the LGM.

## Conclusion

The present study shows deep geographical differentiations of *A. morrisonia*. The genetic distinction among geographical groups is associated with the complicated topology of south China, where high genetic diversity might be conserved and gene flows be blocked. Ecological barriers might result in variant divergence time between two insular groups (Taiwan and Hainan) from China mainland. Isolation by distance seems to be an important factor for genetic structure formation within the geographical populations. Recent demographical expansions corresponding to vegetation changes may have occurred during the largest glacial extension stages rather than the LGM, and more extensive in the eastern part of south China. However, the results suggest a long evolutionary history for *A. morrisonia*, the common ancestor of which could be dated to the late Miocene, and the population differentiations correspond to a series of geological events beyond the Pleistocene ice ages.

## Methods

### Sampling and molecular data

**Figure 3:**
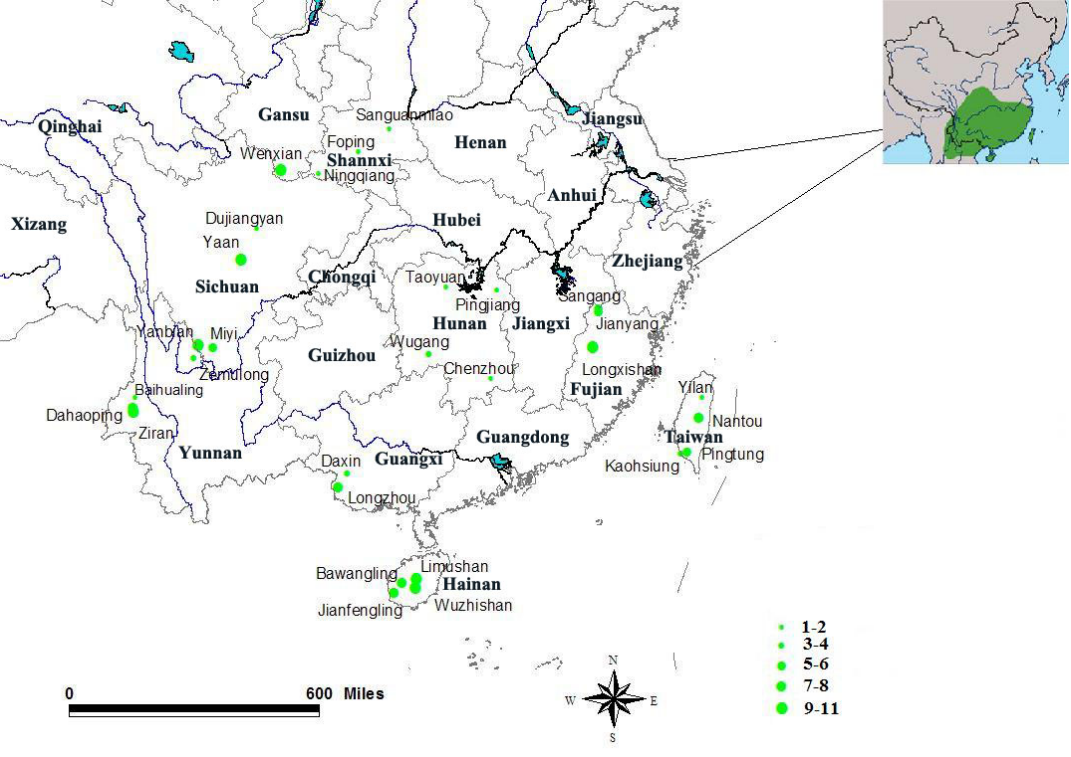
**Sampling sites for *A. morrisonia***. Green segments with plain text represent the sampling locations and sample sizes, while the black text indicates provinces in south China. The small map in the right upper section shows the distribution range of *Alcippe morrisonia *according to Mackinnon et al.

One hundred and sixty birds were collected from 29 localities during 2004 to 2007, covering most of the distribution range of the *A. morrisonia *(Figure 3). Total genomic DNA was extracted from blood or tissue samples using the QIAamp DNA Mini Kit (QIAGEN) following the manufacturer's instructions. A partial cytochrome b (Cytb) gene was amplified with the primer pair OSCL1 (5'-ATGGCCCTCAATCTACGTAAA-3') and OSCH2 (5'-ATAGGACTAGGATGATTGTGAAGTA-3'). The thermocycling program consisted of an initial denaturation at 94°C for 5 min, followed by 40 cycles of 94°C for 40 s, 53°C for 40 s and 72°C for 40 s, plus a final extension at 72°C for 5 min. The same primers were used in sequencing reactions with a Big Dye Terminator Cycle Sequencing Kit v.2.0 and run with an ABI 377 automatic sequencer. The other mitochondrial gene fragment, partial cytochrome c oxidase I (COI), was amplified and sequenced following Hebert *et al*. [56].

Sequences were assembled using Seqman II (DNASTAR) and proofread against the original chromatograms. The presence of stop codons or indels, which could reveal pseudogene sequences, was checked in MEGA3.1 [57]. Sequences were combined by eye and relevant sequences of *Yuhina flavicollis *(EU447103, EU447058), *Alcippe dubia *(FJ754289, FJ754291), *Alcippe brunnea *(FJ754290, FJ754292), *Stachyris ruficepes *(EU447106, EU447061) and *Garrulax sannio *(EU447086, EU447041) were used as outgroups.

### Phylogenetic analysis

Haplotypes for Cytb, COI and the combined sequence were generated in Dnasp, version 4.0 [58]. Maximum parsimony (MP), maximum likelihood [56] and Bayesian inference (BI) phylogenetic analyses were used to identify major clades and to evaluate the relationships among haplotypes of Cytb and COI separately and combined. Modeltest 3.6 [59] and the Akaike information criterion [60] were used to identify the appropriate nucleotide substitution models and the selected models of sequence evolution were used for ML phylogeny reconstruction. MP analyses were performed in PAUP* 4.10b [61] using a heuristic search with 1000 random sequence repetitions and tree-bisection-reconnection (TBR) branch-swapping. ML analyses were performed using PHYML [62]. Non-parametric bootstrapping (1000 replicates) performed in the programs PAUP* 4.10b (MP) and PHYML was used to evaluate nodal support among branches, with 70% or more considered to provide strong support [63]. Bayesian analyses were performed with MrBayes 3.1 [64] with default parameters, using the three selected models generated by Modeltest 3.6 for each gene and the combined dataset. Two independent parallel runs of four incrementally heated Metropolis-coupled MCMCs (Monte Carlo Markov Chains) were run with trees sampled every 100 generations for 5 * 10^6 ^generations or more until to the average standard deviation of split frequency below 0.01. The first 10% of the generations were discarded as 'burn-in', and posterior probabilities were estimated for the remaining saved generations.

### Population genetic analysis

The numbers of haplotypes (*H*), and values of haplotype diversity (*h*) [65] and nucleotide diversity [66] for each sample site, were computed on the basis of the combined sequence dataset in Dnasp, version 4.0. A hierarchical analysis of molecular variance (AMOVA) was performed using pairwise differences; a measure of the extent of DNA divergence between populations was calculated, and the significance was tested using 1,000 permutations with Arlequin version 3.1 [67]. The correlations between genetic and geographic distances were tested by both the Mantel test [68] in Arlequin and in the isolation by Distance Web Service [69].

A maximum parsimony network was constructed using TCS 1.21 [70] with a 95% connection limit. Loops were resolved following the criteria given by Pfenninger and Posada [71]. Haplotypes were hierarchically nested to visualize higher-order patterns of association [72,73]. The null hypothesis of no geographical associations between tip and interior clades was tested using nested clade analysis (NCPA) implemented in Geodis 2.0 [74]. For those clades in which the null hypothesis of random geographical distribution was rejected, potential geographical associations were inferred by the inference key (, updated November 2005).

### Population demographic history

MDIV [75] was used to estimate the divergence time and migration rate between groups. The program uses a Bayesian approach to estimate population divergence times and migration rates simultaneously between pairs of populations that are assumed to have diverged from a common ancestral population. MDIV was run multiple times with different random seeds in order to obtain consistent distributions of results using the following setting: HKY model with the transition/transversion ratio estimated directly from the data; Markov chain simulation for 5,000,000 steps, of which the first 500,000 were discarded as burn-in; and prior distributions from 0 to 10 for *M *and from 0 to 5 for *T*. The divergence times of splits between phylogroup pairs were estimated using the Formula *t*_*divergent time *_= *T*_pop _*(Theta/2 μk) with mutation rate μ and a generation time of 2 years.

Values of Tajima's *D *[76] and Fu's *F *[77] were calculated and used to assess evidence of population expansion for the geographical groups arranged by AMOVA partitions and phylogenetic topology. Mismatch distributions were calculated and the sum of squared deviations (SSD) and raggedness indices (*r*) between observed and expected mismatch distributions were used as a test statistic; their *P *values represented the probability of obtaining a simulated sum of squared deviation greater than or equal to the one observed. Estimation and testing were done by bootstrap resampling (10,000 replicates) using Arlequin 3.1. The relationship Tau = 2 μkt [77] was used to estimate the time of expansion (t), where k is the number of nucleotides assayed and μ is the mutation rate per nucleotide. Because no direct calibration point refers to mutation rate of *A. morrisonia *or its relatives, we cautiously applied "2%"rule of molecular clock for avian Cytb gene [78-82]. An average mutation rate of 1.00*10^-8 ^per site per year for the avian mitochondrial Cytb gene was assumed [9,38]. This mutation rate was modulated by multiplying the ratio of average net distance for the combined sequence *vs. *that for Cytb alone for the geographical group pairs [38]. A generation time of two years was used for *A. morrisonia *according to Sibley & Ahlquist [83] and Zhou, Liu and Li (personal communication).

In order to estimate the dynamics of population size fluctuations over time, we used the Bayesian Skyline Plot (BSP) [84] method implemented in the program BEAST 1.4.6 [85]. This Bayesian approach incorporates the uncertainty in the genealogy by using MCMC integration under a coalescence model, in which the timing of dates provides information about effective population sizes over time. Chains were run for 50 million generations, and first 10% was discarded as 'burn-in'. The substitution model used was HKY+G+I, as selected in Modeltest 3.6. In addition, the times to the most recent common ancestor (T_MRCA_) of the seven geographical groups and the whole population were estimated using the same mutation rate as above. The results were summarized through TRACER 1.4 (Rambaut & Drummond 2007, Available from ).

## Authors' contributions

This work is part of GS's Ph.D. thesis. GS is a Ph.D. graduate student of FL. He carried out and designed the study. He accomplished the laboratory work and statistical analyses with guidance and help from YQ, and drafted the manuscript. ZY participated in most of the field sample collecting. SL coordinated the study in sequencing samples from Taiwan and revised the draft of the manuscript. NL improved the organization of the discussion and corrected the choice of generation time for *A. morrisonia*. FL conceived and elaborated the design of the whole study, including sample collection, preparation, analysis and revision of the manuscript. All authors read and approved the final manuscript.

## Supplementary Material

Additional file 1 **Genetic variability and haplotypes based on 1236 bp of combined mitochondrial sequences of *Alcippe morrisonia***. A summary of genetic variability in each sample location based on combined mitochondrial data. Sample size (n), number of haplotypes (H), haplotype diversity (h), nucleotide diversity (π). Haplotype label in bold indicate it was shared in different samples and bold with * indicates it was shared between different locations. Click here for file

Additional file 2 **AMOVA analysis of *A. morrisonia***. AMOVA result shows that most of the variance came from differences among groups, and the seven groups were best recognized with the maximum value of among-group variance. Click here for file

Additional file 3 **Genetic diversity and mismatch distribution analysis of the geographical groups**. The genetic diversity and mismatch distribution for seven geographical groups were summarized in the table. *N*, group size, *H *and *π *is the genetic diversity index. *P*_*SSD *_and *P*_*H*-*R *_are parameters of the goodness-of-fit test to the sudden expansion model. *Tau *is the time in number of generations elapsed since the sudden expansion. *T *is the expansion time transformed by Tau = 2 μkt. Click here for file

Additional file 4 **Nested clade phylogeographical analysis with IBD tests for *A. morrisonia***. NCPA results show Allopatric fragmentation in network A. For low level clades, restricted gene flow with isolation by distance was revealed. However these associations were not confirmed by the Mantel Test or IBDWS; only clade 4-1 in network A showed a tendency towards a significant IBD pattern. Click here for file
