# Supplementary material for: Phylogeography of the Alcippe morrisonia (Aves: Timaliidae): long population history beyond late Pleistocene glaciations
Source: BMC Evol Biol. 2009 Jun 27;9:143. doi: 10.1186/1471-2148-9-143 (PMC2714695; doi:10.1186/1471-2148-9-143)
Supplement: Additional file 1 — Genetic variability and haplotypes based on 1236 bp of combined mitochondrial sequences of Alcippe morrisonia. A summary of genetic variability in each sample location based on combined mitochondrial data. Sample size (n), number of haplotypes (H), haplotype diversity (h), nucleotide diversity (π). Haplotype label in bold indicate it was shared in different samples and bold with * indicates it was shared between different locations. [file 1471-2148-9-143-S1.doc]

# Additional files

Additional file 1

|  | **Geogroup belonging to** | ***n*** | ***H*** | ***h*** | ***π* (%)** |  |
| --- | --- | --- | --- | --- | --- | --- |
| **Chenzhou** | Fujian | 2 | 3 | 1 | 0.243 | **H1***, H2 |
| **Longxishan** | Fujian | 10 | 10 | 1 | 0.552 | H18, H19, H38, H38, H39, H40, H41, H42, H43, H44, |
| **Jianyang** | Fujian | 5 | 5 | 1 | 0.518 | **H1***, H20, H21, H45, H46 |
| **Sangang** | Fujian | 6 | 4 | 0.867 | 0.270 | **H22**, **H47**, H48, H49 |
| **Pingjiang** | Fujian | 1 | 1 | / | / | H26 |
| **Limushan** | Hainan | 10 | 7 | 0.911 | 0.230 | H84, H85, H86, **H87***, **H88***, **H89***, **H90** |
| **Wuzhishan** | Hainan | 10 | 7 | 0.911 | 0.201 | **H88***, **H89***, H91, H92, H93, H94, H95 |
| **Jianfengling** | Hainan | 8 | 7 | 0.964 | 0.445 | **H87***, H96, **H97**, H98, H99, H100, H101 |
| **Bawangling** | Hainan | 8 | 7 | 0.964 | 0.318 | **H88***, H102, H103, **H104**, H105, H106, H107 |
| **Nantou** | Taiwan | 4 | 4 | 1 | 0.337 | **H108***, H115, H116 |
| **Pingtung** | Taiwan | 4 | 3 | 0.833 | 0.202 | H83, **H110**, H111, H114 |
| **Kaohsiung** | Taiwan | 4 | 4 | 1 | 0.364 | **H108***, H109, H112, H113, |
| **Baihualing** | WYunnan | 1 | 1 | / | / | H58 |
| **Dahaoping** | WYunnan | 8 | 7 | 0.964 | 0.717 | **H59**, H60, H61, **H62***, H63, H64, H65 |
| **Ziran** | WYunnan | 10 | 7 | 0.911 | 0.624 | **H62***, **H66**, H67, H68, H69, H70, H71 |
| **Miyi** | SWSichuan | 5 | 3 | 0.8 | 0.081 | **H7**, H8, **H9*** |
| **Zemulong** | SWSichuan | 4 | 3 | 0.833 | 0.081 | **H9***, H12, H13, H14, H72, H73, H74 |
| **Yanbian** | SWSichuan | 10 | 7 | 0.867 | 0.649 | **H9***, H27, H28 |
| **Wugang** | Centre | 4 | 3 | 0.833 | 0.094 | H3, H4, **H25** |
| **Dujiangyan** | Centre | 2 | 2 | 1 | 0.485 | H5, H6 |
| **Yaan** | Centre | 9 | 9 | 1 | 0.506 | H10, H11, H29, H30, H31, H32, H33, H34, H35 |
| **Foping** | Centre | 1 | 1 | / | / | H15 |
| **Ningqiang** | Centre | 1 | 1 | / | / | H16 |
| **Sanguanmiao** | Centre | 2 | 2 | 1 | 0.405 | H17, H36 |
| **Wenxian** | Centre | 11 | 9 | 0.964 | 0.377 | H23, **H50**, H51, H52, H53, H54, **H55**, H56, H57 |
| **Taoyuan** | Centre | 1 | 1 | / | / | H24 |
| **Longzhou** | Guangxi | 7 | 6 | 0.952 | 0.57 | **H75**, H76, H77, H78, H79, H80 |
| **Daxin** | Guangxi | 3 | 3 | 1 | 0.27 | **H81**, H82 |
|  |  |  |  |  |  |  |
| **Total** |  | 151 | 116 | 0.9934 | 5.024 |  |
