# Supplementary material for: Phylogeography of the Alcippe morrisonia (Aves: Timaliidae): long population history beyond late Pleistocene glaciations
Source: BMC Evol Biol. 2009 Jun 27;9:143. doi: 10.1186/1471-2148-9-143 (PMC2714695; doi:10.1186/1471-2148-9-143)
Supplement: Additional file 2 — AMOVA analysis of A. morrisonia. AMOVA result shows that most of the variance came from differences among groups, and the seven groups were best recognized with the maximum value of among-group variance. [file 1471-2148-9-143-S2.doc]

**Additional file 2**

| **Source of variation** | **Sum of squares** | **Variance components** | **Percentage of variation** | **Fixation indices** | ***P*** |
| --- | --- | --- | --- | --- | --- |
| Among groups | 4192.657 | 33.1268 | 90.92 | ***Φ***_CT_:0.9091 | 0.0000 |
| Among populations within groups | 78.644 | 0.1137 | 0.31 | ***Φ***_SC_:0.0343 | 0.0635 |
| Within populations | 392.971 | 3.1948 | 8.77 | ***Φ***_ST_:0.9123 | 0.0000 |
