# Supplementary material for: Phylogeography of the Alcippe morrisonia (Aves: Timaliidae): long population history beyond late Pleistocene glaciations
Source: BMC Evol Biol. 2009 Jun 27;9:143. doi: 10.1186/1471-2148-9-143 (PMC2714695; doi:10.1186/1471-2148-9-143)
Supplement: Additional file 3 — Genetic diversity and mismatch distribution analysis of the geographical groups. The genetic diversity and mismatch distribution for seven geographical groups were summarized in the table. N, group size, H and π is the genetic diversity index. PSSD and PH-R are parameters of the goodness-of-fit test to the sudden expansion model. Tau is the time in number of generations elapsed since the sudden expansion. T is the expansion time transformed by Tau = 2 μkt. [file 1471-2148-9-143-S3.doc]

**Additional file 3**

| **Group** | ***n*** | ***H*** | ***π* (%)** | **Tajima's D test** | | **Fu's Fs** | | **Mismatch distribution Analysis** | | | |
| --- | --- | --- | --- | --- | --- | --- | --- | --- | --- | --- | --- |
|  |  |  |  | ***Tajima's D*** | ***P*** | ***FS*** | ***P*** | ***P_SSD_*** | ***P****_H-R_* | ***Tau* (CI=95%)** | ***T*（Mya）** |
| **Fujian** | 24 | 0.9891 | 0.54 | -1.3265 | 0.0760 | -12.7723 | 0.0000 | 0.29 | 0.04 | 2.014(0.855-14.525) | 0.18(0.08-1.31) |
| **Hainan** | 36 | 0.9555 | 0.33 | -1.9401 | 0.0120 | -16.6975 | 0.0000 | 0.24 | 0.36 | 3.699(1.953-5.166) | 0.33(0.18-0.46) |
| **Taiwan** | 12 | 0.9697 | 0.31 | -1.2431 | 0.1020 | -4.7870 | 0.0090 | 0.46 | 0.52 | 4.312(1.627-7.561) | 0.39(0.15-0.68) |
| **WYunnan** | 19 | 0.9532 | 0.65 | -1.1433 | 0.1190 | -3.1143 | 0.0880 | 0.35 | 0.19 | 9.967(5.307-16.012) | 0.89(0.47-1.44) |
| **SWSichuan** | 19 | 0.8304 | 0.39 | -2.3971 | 0.0000 | -2.1404 | 0.1590 | 0.00 | 0.96 | 0.648(0.000-1.391) | 0.06(0.00-1.25) |
| **Centre** | 31 | 0.9935 | 0.38 | -1.9446 | 0.0100 | -25.5709 | 0.0000 | 0.39 | 0.36 | 4.844(3.510-6.154) | 0.43(0.32-0.55) |
| **Guangxi** | 10 | 0.9555 | 0.48 | -0.2988 | 0.3990 | -1.5268 | 0.1540 | 0.28 | 0.52 | 1.068(0.000-6.045) | 0.09(0.00-0.54) |
