# Supplementary material for: Phylogeography of the Alcippe morrisonia (Aves: Timaliidae): long population history beyond late Pleistocene glaciations
Source: BMC Evol Biol. 2009 Jun 27;9:143. doi: 10.1186/1471-2148-9-143 (PMC2714695; doi:10.1186/1471-2148-9-143)
Supplement: Additional file 4 — Nested clade phylogeographical analysis with IBD tests for A. morrisonia. NCPA results show Allopatric fragmentation in network A. For low level clades, restricted gene flow with isolation by distance was revealed. However these associations were not confirmed by the Mantel Test or IBDWS; only clade 4-1 in network A showed a tendency towards a significant IBD pattern. [file 1471-2148-9-143-S4.doc]

**Additional file** 4

| Clade | Clues | Inference | Mateltest | | IBDWS | |
| --- | --- | --- | --- | --- | --- | --- |
|  |  |  | *r* | *P* | *r* | *P* |
| A |  |  |  | |  | |
| 2-12 | 1-2-3-4:No | Restricted gene flow with isolation by distance | 0.7171 | 0.3080 | 0.2651 | 0.5330 |
| 3-1 | 1-2-11-17:No | Inconclusive outcome |  | |  | |
| 4-1 | 1-2-11-17-4:No | Restricted gene flow with isolation by distance | 0.6232 | 0.0810 | 0.6232 | 0.1090 |
| 4-2 | 1-2-3-4:No | Restricted gene flow with isolation by distance | 0.6505 | 0.1690 | 0.2187 | 0.3000 |
| Total | 1-19-20:No | Allopatric Fragmentation |  | |  | |
| C |  |  |  | |  | |
| 4-1 | 1-2-11-17:No | Inconclusive outcome |  | |  | |
| D |  |  |  | |  | |
| 2-1 | 1-2-3-4:No | Restricted gene flow with isolation by distance | -0.00001 | 0.5270 | -0.0001 | 0.5280 |
| Total | 1-19-20:No | Inadequate geographical sampling |  | |  | |
